# Improving Islet Engraftment by Gene Therapy

**DOI:** 10.1155/2011/594851

**Published:** 2011-10-24

**Authors:** Xiaojie Wang, Mark Meloche, C. Bruce Verchere, Dawei Ou, Alice Mui, Garth L. Warnock

**Affiliations:** ^1^Department of Surgery, University of British Columbia, Vancouver, BC, Canada V5Z 4E3; ^2^Department of Pathology and Laboratory Medicine, University of British Columbia, Vancouver, BC, Canada V5Z 4H4

## Abstract

Islet cell transplantation is currently the only feasible long-term treatment option for patients with type 1 diabetes. However, the majority of transplanted islets experience damage and apoptosis during the isolation process, a blood-mediated inflammatory microenvironment in the portal vein upon islet infusion, hypoxia induced by the low oxygenated milieu, and poor-revascularization-mediated lack of nutrients, and impaired hormone modulation in the local transplanted site. Strategies using genetic modification methods through overexpression or silencing of those proteins involved in promoting new formation of blood vessels or inhibition of apoptosis may overcome these hurdles and improve islet engraftment outcomes.

## 1. Introduction

Islet cell transplantation has been recognized as a treatment option for Type 1 diabetes (T1D) after the introduction of the Edmonton protocol that emphasizes the requirement for both an adequate amount of donor islets and the use of steroid-free immunosuppressive regimens [1]. However, international trials conducted around the world demonstrate a constant decline of graft function and a relatively low rate of successful engraftment compared with other solid organ transplantations [2–5].

Islet cell transplantation has a much poorer success rate than whole pancreas transplantation despite the same degree of mismatched major histocompatibility complex (MHC) alleles and similar immunosuppressive treatment protocols in both types of pancreatic transplantation. The pancreas survival (insulin-independent) rate for whole organ transplantation was 90%, 67%, and 36% at 1, 5, and 15 years respectively, after transplantation [6]. By contrast, the insulin independent survival rate for pancreatic islet cell transplantation rate dropped sharply to 10% at 5-years despite comparable success rates at one year in both types of transplantation [7]. 

Several factors may explain the discrepancies between successful long-term pancreas engraftment and the low rate of success in islet transplantation. The key difference between the two approaches is the mode of blood supply reestablishment. Similar to other solid organ transplantations, the transplanted pancreas can receive immediate blood supply via physical reconnection of arterial and venous vessels. By contrast, islets are avascular for several days following transplantation, and blood flow to transplanted islets is generated through angiogenesis and/or vasculogenesis thereafter [8–11]. 

Reconnection of blood supply is not only slower for islet cells, but also inferior compared with whole pancreas transplants. Data indicate that transplanted islets are less vascularized and have a lower oxygen tension than naïve islets before isolation; even after revascularization is complete [8–11]. As such, up to 70% of transplanted beta cells' mass may be destroyed in the early islet posttransplant period in both immunodeficient and syngeneic transplantation models, suggesting a critical role of nonimmune factors in determining the outcome of islet transplantation [12, 13]. Therefore, it is very important to address the nonimmune aspects as well as allo- and/or auto-immune-mediated graft loss in islet transplantation to maximize the usage of limited donor resources. 

One of the fundamental requirements for successful islet cell transplantation is the infusion of sufficiently large quantities of islets. Although it only requires as low as 20% of the islets within a pancreas to normalize the hyperglycemic state in diabetic cohorts, there are significant challenges to achieve satisfactory clinical outcomes in terms of insulin independence. Key adverse factors that limit successful islet transplantation include tissue damage and cell apoptosis induced during the islet isolation process, acute blood-mediated inflammatory injury of islets injected into the portal vein, and extreme cytotoxicity to beta cells due to high concentration of immunosuppressant drugs accumulated in the transplanted site in addition to the above-mentioned hypoxia and prolonged oxidative stress caused by slow and/or poor revascularization. Addressing these issues will be instrumental for improving successful rates of islet engraftment in clinical islet cell transplantation [8, 14–19]. 

The strategies for improving islet engraftment could be achieved in several ways based on their origins. One of the required procedures for islet transplantation is the isolation of donor pancreas into individual islet cells. This step potentially allows for genetic modification *ex vivo* to improve the survival of isolated islets, to increase the beta mass, and to speed the process of new blood vessel reestablishment for supply of oxygen and nutrients. In this review, we will highlight the aspects of improving islet survival via gene overexpression or deletion to minimize apoptosis during isolation, to promote angiogenesis for revascularization, and to avoid blood-mediated inflammatory responses.

## 2. Structure and Function of Islets of Langerhans

**Figure 1:**
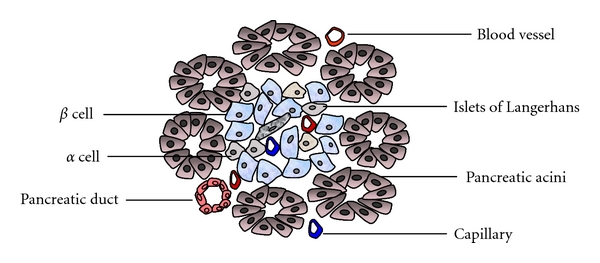
Structure of the pancreas. Pancreas tissue consists of both exocrine and endocrine glands. Islets of Langerhans mainly include *α*-, *β*-, and *δ*-cells. The islets are arranged in clusters associated with a dense network of capillaries. Most islet cells are directly in contact with blood vessels.

Islets or islets of Langerhans are vascularized clusters of cells within the pancreas that contain the insulin-producing *β* cells. They were named after Paul Langerhans who discovered islets in 1869 [4]. There are five types of cells within an islet, *α*, *β*, *δ*, pp, and *ε* cells that secrete hormones of glucagon, insulin, somatostatin, pancreatic polypeptide, and ghrelin, respectively. These cells reside in groups that Langerhans likened to small islands in the pancreas and release different hormones into the portal vein in response to changes in blood nutrient levels (Figure 1). Destruction of insulin producing *β* cell by autoreactive T cells causes type 1 diabetes. Restoration of *β*-cell mass by islet transplantation into the liver through the portal vein is considered to follow the physiological route for hormone release.

The average weight of a human pancreas is 68 g. The size of an islet ranges from 50 to 250 *μ*M, with a mean diameter of 140 *μ*M. The average numbers of islets for normal humans are approximately 1 million, with a total beta-cell mass of 500 to 1500 mg. The islets of Langerhans constitute 1-2% of pancreas mass. However, they receive 10–20% of pancreatic arteriolar flow, demonstrating a significant need for oxygen and nutrients for immediate insulin secretion in response to glucose stimulation [20, 21]. 

The distribution and percentage of individual cell types in the islet are species-specific. Insulin expressing *β* cells are the most abundant in all species, but the proportion and localization are not the same [20, 21]. The percentage of insulin positive cells is lower in human (55%) than in mouse islets (77%). Those insulin-containing cells are located in the core of each mouse islet. On the contrary, in human islets, they are intermingled with other types of cells. The glucagon secreting *α* cells are fewer (20%) compared with *β* cells and located in the periphery of the mouse islets. By contrast, they are only slightly lower in number than *β* cells (40%) and scattered throughout the human islets. These discrepancies result in a higher *β*/*α* cell ratio in mice and *β* cells form the inner core of the mouse islets as compared to humans. In the human pancreas, the other cell types are located predominately in the periphery with the exception of somatostatin-positive cells that are present throughout the islet [21]. 

Despite the distinct cytoarchitecture and composition of different cell types cross-species, all cell types reside close to islet blood vessels; no portion of an islet is more than one cell away from arterial blood [9]. Approximately 86% of the *α* cells and 77% of the *β* cells are closely apposed to vascular endothelial and smooth muscle cells [21]. Although the different cell types are aligned along the vessels with no particular order, most *β* cells face *α* and *δ* cells across the blood vessel lumen [21]. The strategies for an islet to survive and fulfill its function include enough contact with the blood vessels and structural permeability to ensure sufficient flow of arterial blood into each individual islet (Figure 1). Arterioles enter islets and form fenestrated microcirculation networks similar to renal glomerular-like structures to allow for bidirectional endocrine exchange and blood for nutrients and oxygen [9]. An islet develops an intensive network of capillaries with a thin collagen capsule and glial sheet that separates the endocrine from the exocrine pancreas. In order to regulate hormone secretion and function, islets are also abundantly innervated by sympathetic and parasympathetic nerves. 

This complex structural arrangement is thought to maintain glucose homeostasis through providing enough supply of oxygen and nutrients to the islet cells, to enable their metabolic sensing, and to allow the rapid distribution of secreted hormones to target organs. However, this perfect association is severely disrupted after pancreatic islets are isolated for transplantation. The essential nutrients and oxygen for islet survival and normal function can only be supplied through diffusion from the surrounding tissue into the transplanted islets following transplantation. A new islet vascular system can be formed within 7–14 d [10, 11, 22]. However, the regenerated islet vascular network is not the same as that observed in islets before transplantation. In mice, the regenerated network is only one third of that seen in naïve islets, even when revascularization is complete [23]. Similarly, engraftment is low when human islets are transplanted to nude mice intraportally to the liver or at the renal site [17, 24].

## 3. Interventions to Improve Graft Vascular Function

The majority of transplanted islets are lost during the early stages of transplantation; only approximately 20 to 30% of donor islets are stably engrafted [22–24]. The reasons are partially due to the disconnection of blood supply during islet isolation and the slow/poor revascularization process after islet transplantation. Therefore, islet engraftment rates might be improved if interventional strategies for promoting newly formed blood vessels are employed. 

The growth of new blood vessels from preexisting vessels or so called angiogenesis is a multiple-step event that includes pericellular proteolysis, sprouting or migration/proliferation of endothelial cells (EC), formation of new capillaries, and maturation of blood vessels. The sequence of molecular events for angiogenesis in islet transplantation is largely unknown. However, it has been shown that recruitment of angioblasts and overexpression of vascular endothelial growth factor (VEGF) in the isolated islets can promote angiogenesis and improve islet graft function [25–27]. Three key families of angiogenic factor receptor tyrosine kinases, the VEGF receptors, Tie receptors, and Eph receptors play major roles in regulating the process of angiogenesis [28, 29]. VEGF is a key regulator of physiological angiogenesis during embryogenesis, skeletal growth, and reproductive functions. The absolute requirement for VEGF in blood vessel development is well established in mice. Mice with single VEGF allele mutation die before birth as a result of abnormal blood vessel formation, demonstrating strict dependence on VEGF during development [30, 31]. 

VEGF-A is secreted by *β*-cells and plays a critical role in regulating islet vascularization and function. It is also a major regulator for interaction between *β*- and endothelial-cells [32–34]. Several lines of evidence support this notion. First, *β*-cell-specific inactivation of VEGF-A in mouse studies leads to reduced islet blood vessel density and size without affecting the exocrine vascular system. The substantial loss of islet vessels, density, size, and permeability causes less blood flow into the mutants' islets. Therefore, these mice have impaired glucose-stimulated insulin secretion (GSIS) resulting from decreased overall flux of the stimuli into the islets and a lower/delayed *β*-cell response following *β*-cell exocytosis [32–34]. Further investigation revealed that insulin deficiency is not a result of *β*-cell dysfunction, but rather it is caused by abnormalities in islet vasculature. In summary, mice deficient for VEGF-A in pancreatic islets exhibit abnormal phenotypes of both reduced vasculature and impaired glucose tolerance. 

VEGF-A is also required for vascularization of transplanted islets, but the process of isolation and transplantation results in reduced expression of VEGF-A [27, 28]. Overexpression of VEGF-A via adenoviral transduction in isolated islets, or endogenous expression in transgenic mice, results in superior transplantation outcomes [27, 28]. VEGF-A is normally expressed in islets *in situ* but expression levels decrease 2-3 days after transplantation [27, 28]. Islets deficient in VEGF-A exhibit inefficient revascularization after transplantation into normal hosts and extensive islet cell death immediately after transplantation [32–34]. Both host blood vessels and residue donor islet endothelial cells participate in the formation of a new islet vascular network for graft vessel formation [22, 35, 36]. Reduced expression of VEGF-A results in not only decreased numbers of intraislet endothelial cells in donor islets, but also inadequate recruitment of host endothelial cells and their migration into the grafts [32–34]. Loss or gain of function by genetic manipulation shows that VEGF-A is not only essential for significant angiogenic capacity, but also necessary for vascularization. Endogenous expression VEGF-A from transgenic mice under the control of a rat insulin promoter, or transient overexpression via adenoviral delivery, significantly enhances microvascular density, functional blood flow to the graft, and long-term survival of functional islet mass [26, 27].

**Figure 2:**
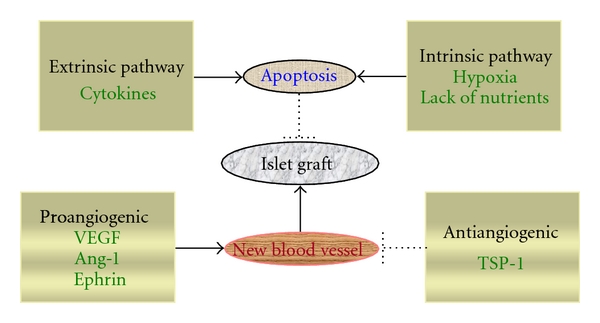
Factors controlling successful islet engraftment. The successful engraftment of transplanted islets is determined by both the ability to form new blood vessels and the capacity to limit apoptosis induced by both extrinsic (induced by extracellular inducers, such as inflammatory cytokines) and intrinsic (induced by intrinsic factors, such as hypoxia and nutrient deprivation) pathways. Proangiogenic factors such as VEGF, Ang-1, and ephrin promote angiogenesis. By contrast, antiangiogenic factors such as TSP-1 reduce this process.

Under normal physiological conditions, vasculature and/or endothelial cells proliferate at a very low rate. This quiescence is thought to be maintained by the balance between proangiogenic (also called stimulators) and antiangiogenic factors (inhibitors). Therefore, strategies for promoting new blood vessel formation include both overexpression of stimulators and deletion of inhibitors (Figure 2). Thrombospondin-1 (TSP-1) is an inhibitor of angiogenesis [37–40]. Its antiangiogenic function is through the regulation of extracellular matrix function, blood clot formation, and immune responses [37–40]. The unique feature of inhibitory regulation by TSP-1 is its selective induction of apoptosis on activated, but not quiescent, endothelial cells through binding to their extracellular matrix, suggesting a highly selective target to newly forming blood vessels without disturbing other nondividing endothelial cells [37]. In addition, it also functions as an antagonist of angiogenesis by preventing the recruitment of proangiogenic factors, such as matrix metalloproteinase-9 and VEGF, to the site for new blood-vessel formation, and blocking the binding to their counter-receptors on the endothelial cells [41]. In concordance with this function, TSP-1 knockout mice have hypervascularized islets [37]. Islets made deficient in TSP-1, through knockout gene expression or RNA silencing, display an increased vascular density when compared with control islets 1 month after transplantation [42]. However, subsequent studies revealed that TSP-1 is important for *β* cell function [43]. Mice deficient in TSP-1 show glucose intolerance, impaired insulin biosynthesis, and glucose oxidation rate. TSP-1 deficient mice treated with transforming growth factor (TGF) *β*-1-activating sequences recover their normal capacity of glucose tolerance, suggesting that TSP-1 functions via the TGF*β*-1 pathway [43]. Deletion of antiangiogenic factor TSP-1 promotes islet engraftment through creating a microenvironment for blood vessel growth, blood perfusion and oxygenation in the graft, therefore, improving islet graft revascularization and function.

## 4. Strategies to Prevent *β*-Cell Apoptosis

Apoptosis induced in the transplanted islets arises from the isolation process, a blood-mediated inflammatory reaction upon injection, immunosuppressive drug-related direct toxicity to *β*-cell function, and hypoxia/oxidative stress during the revascularization process [4, 5, 16].

Programmed cell death or apoptosis can be regulated through two distinct pathways, namely, the extrinsic and intrinsic pathways (Figure 2). In the extrinsic pathway, extracellular events, such as the release of inflammatory cytokines during the disassociation and isolation of donor islets via collagenase/protease digestion, can initiate *β*-cell apoptosis. Proapoptotic cytokines and other signaling molecules activate apoptosis initiator caspase 8, leading to effector caspase activation, and promoting cleavage of procaspase to activated caspase 3. Intracellular or intrinsic inducers, such as hypoxia and nutrient deprivation due to disconnection of the blood supply during donor islet isolation and the revascularization process, activate the mitochondrial pathway. This pathway is regulated by the balance between proapoptotic proteins, such as Bax and Bad, and anti-apoptotic proteins, such as Bcl-2 and Bcl-XL. The release of cytochrome c from mitochondria to the cell cytosol stimulates the activation of apoptosis initiator caspase 9, which then activates effector caspase 3. Strategies for preventing *β*-cell apoptosis are considered in three different ways: blocking the inducers, modulating key proteins in each pathway, or inhibiting effector caspases at the final stages [44].

The inhibitor of apoptosis protein (IAP) family includes a number of endogenous antiapoptotic genes, including X-linked IAP (XIAP). XIAP is the most potent caspase inhibitor in the IAP family and functions to restrain apoptotic cell death by preventing activation of initiator caspase 9, as well as effector caspases 3 and 7, through binding to the active site of those caspases. Overexpression of XIAP in donor islets dramatically reduces the number of human islets required to reverse hyperglycemia in chemically diabetic immunodeficient mice [45]. It also reverses the adverse effects of immunosuppressive drugs on insulin secretion and enhances cell viability of isolated human islets [46]. 

Another apoptosis inhibitor also shows beneficial effects on preventing *β*-cell apoptosis. Treatment with Val-Pro-Met-Leu-Lys (V5), a cell-permeable apoptosis inhibitor pentapeptide, improves the donor islet mass following collagenase digestion and isolation [47]. This treatment also reduces the number of donor islets required for reverse hyperglycemia in streptozotocin-induced diabetic mice. The mechanism for this action is through upregulating anti-apoptotic proteins Bcl-2 and XIAP as well as downregulating Proapoptotic proteins Bax and Bad. These studies indicate that the inhibition of apoptosis by overexpression of anti-apoptotic proteins directly or indirectly significantly improves islet function following isolation and improves islet graft function post transplant. 

*β* cells are known to be sensitive to apoptosis. Several mediators have been found to cause dysfunction and/or cellular death of the pancreatic islets including inflammatory cytokines such as interleukin 1 *β* (IL-1*β*), TNF-*α*, and IFN-*γ*, which in turn promote the release of inducible nitric oxide synthase (iNOS) and cytotoxic nitric oxide (NO) [48–51]. In fact, the production of iNOS and NO is significantly elevated after transplantation [51]. Blocking of iNOS via small interfering RNA (siRNA) decreases iNOS expression, NO production, and iNOS/NO-mediated apoptosis [52]. Inflammatory cytokines are also known to be upregulated due to the oxidative stress induced during isolation and nutrient deprivation after transplantation [51]. More importantly, systemic administration of IL-1 receptor antagonist protein subcutaneously or local delivery via adenovirus prevents IL-1*β*-induced *β*-cell impairment/apoptosis *in vitro* and prolongs mouse islet allograft survival *in vivo *[49, 50]. 

The numbers of donor islets required for transplantation could be reduced using reagents that block apoptosis through inhibiting proinflammatory cytokines, or modulating the proteins participating in the extrinsic and/or intrinsic apoptosis pathways. Islet engraftment improvement might also be achieved through increasing *β*-cell mass [53, 54]. Adenoviral delivery of hepatocyte growth factor (HGF) to nonhuman primate islets improves graft survival and function in streptozotocin-induced diabetic NOD-SCID mice, markedly reducing the number of islets required to achieve normal glucose control. The delivery method for up- or down-regulation of the genes to improve islet engraftment has been limited to mostly replication-deficient adenoviral systems due to the nondividing feature of the islet cells. Several studies have explored the nonviral vector for application in clinical islet transplantation [55, 56]. Both Effectene and polyethylenimine showed higher gene-delivery efficiency for pancreatic islets compared with other classes of nonviral delivery systems and are promising as gene delivery agents for pretransplant *ex vivo* gene therapy of islets.

## 5. Conclusion

Islet cell transplantation itself is a great surgical procedure for treating type 1 diabetes. However, it is largely impeded by the limited number of islet donors. In order to achieve insulin independence, at least 10,000 islet equivalents (IE)/kg recipient body weight is needed [1–5]. In fact, significantly more islets must be transplanted to reverse hyperglycemia because a considerably high number of donor islets are lost due to the injuries from enzymatic/mechanical-mediated damage during isolation, and lack of oxygen and nutrient supply during the slow reestablishment of blood supply to the transplanted islets. In another words, if this significant loss of donor islets can be avoided, then more diabetic recipients can be transplanted, and/or better insulin-independence rates will be achieved with the same number of currently available donors. Interventional strategies to improve islet engraftment by gene therapy could have a dramatic impact on the number of patients that might benefit from this therapy and could affect long-term graft survival.

## Abbreviations

EC: Endothelial cells
GSIS: Glucose-stimulated insulin secretion
HGF: Hepatocyte growth factor
IAP: The inhibitor of apoptosis protein
IE: Islet equivalents
IL: Interleukin
iNOS: Inducible nitric oxide synthase (iNOS)
MHC: Major histocompatibility complex
NO: Cytotoxic nitric oxide
siRNA: Small interfering RNA
TGF: Transforming growth factor
TSP-1: Thrombospondin-1
VEGF: Vascular endothelial growth factor
XIAP: X-linked inhibitor of apoptosis protein.
